# Synthesis, Spatial Structure and Analgesic Activity of Sodium 3-Benzylaminocarbonyl-1-methyl-2,2-dioxo-1*H*-2λ^6^,1-benzothiazin-4-olate Solvates

**DOI:** 10.3390/scipharm84040705

**Published:** 2016-10-19

**Authors:** Igor V. Ukrainets, Lidiya A. Petrushova, Svitlana V. Shishkina, Lina A. Grinevich, Galina Sim

**Affiliations:** 1 Department of Pharmaceutical Chemistry, National University of Pharmacy, 53 Pushkinska St., 61002 Kharkiv, Ukraine; dika-l1@ya.ru (L.A.P.); g.l.a-14@yandex.ru (L.A.G.); 2 SSI “Institute for Single Crystal” National Academy of Sciences of Ukraine, 60 Nauki ave., 61001 Kharkiv, Ukraine; sveta@xray.isc.kharkov.com; 3 Department of Inorganic Chemistry, V. N. Karazin Kharkiv National University, 4 Svobody sq., 61077 Kharkiv, Ukraine; 4 Department of Pharmaceutical Chemistry, Far Eastern State Medical University, 35 Murav’eva-Amurskogo St., 680000 Khabarovsk, Russia; sim.hab@mail.ru

**Keywords:** analgesic activity, conformation, conformer, crystal structure, 4-hydroxy-2,2-dioxo-1*H*-2λ^6^,1-benzothiazine-3-carboxamide, solvate

## Abstract

In order to obtain and then test pharmocologically any possible conformers of the new feasible analgesic *N-*benzyl-4-hydroxy-1-methyl-2,2-dioxo-1*H*-2λ^6^,1-benzothiazine-3-carboxamide, its 4-*O*-sodium salt was synthesized using two methods. X-ray diffraction study made possible to determine that, depending on the chosen synthesis conditions, the above-mentioned compound forms either monosolvate with methanol or monohydrate, where organic anion exists in the form of three different conformers. Pharmacological testing of the two known pseudo-enantiomeric forms of the original *N-*benzylamide and of the two solvates of its sodium salt was performed simultaneously under the same conditions and in equimolar doses. Comparison of the results obtained while studying the peculiarities of the synthesized compounds spatial structure and biological properties revealed an important structure-action relationship. In particular, it was shown that the intensity of analgesic effect of different conformational isomers of *N-*benzyl-4-hydroxy-1-methyl-2,2-dioxo-1*H*-2λ^6^,1-benzothiazine-3-carboxamide may change considerably: while low active conformers are comparable with piroxicam, highly active conformers are more than twice as effective as meloxicam.

## 1. Introduction

It is generally known from the organic chemistry course that, due to the rotation of their separate parts around simple bonds, organic molecules can take various space forms called conformational (rotational) isomers or conformers. Apart from pure theoretical interest, this property attracts scientists’ attention by its practical value. Molecule conformation can be shown not only to define its outer form but often its ability to react as well. This is especially important for such complicated reactions as interaction of physiologically active compounds with biological structures sensitive to them—enzymes or receptors—when strict complementarity is of exceptional importance. That is why there is a common opinion in medical chemistry that the biological effect of a lot of compounds depends on their molecules’ ability to exist in the form of specific conformations most propitious for interaction with a proper receptor or enzyme.

A lot of organic compounds are known to produce a clearly marked pharmacological effect only in a strictly fixed conformation. Proteins are especially indicative [1] due to the fact that their denaturation is usually accompanied only by the changes of tertiary or secondary native conformation, which is sufficient to cause total loss of biological function associated to a certain protein, even though the changes do not affect its original structure. Multiple conforms (conformational polymorphism) of pharmaceuticals can often be observed [2]. In contrast to enzymes, change from one pharmaceutical conform to another may be spontaneous or performed under various (unpredictable or just inexplicable) factors [3]. There is no danger if such structural changes do not lead to substantial changes of the pharmaceuticals biological properties. However, if conformers happen to be pharmacologically unsafe (as happened to the HIV drug ritonavir [4] and Parkinson’s disease drug rotigotine [5]), they may be really dangerous to people’s health or even life. It is not surprising, then, that the developers of physiologically active compounds try to study most fully and thoroughly all possible conformations of the feasible medicine before it is launched in the pharmaceutical market [6,7,8,9].

**Figure 1:**
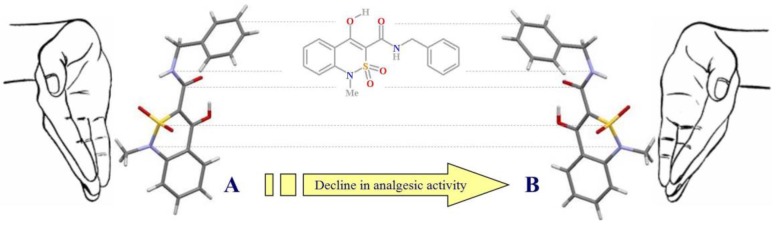
Pseudo-enantiomeric conformers of *N-*benzyl-4-hydroxy-1-methyl-2,2-dioxo-1*H*-2λ^6^,1-benzothiazine-3-carboxamide (**1**) [13].

All possible theoretical calculations are being used to reveal highly or low active conformers [10,11]. However, although these methods have been reported to be successful, they are far from being perfect as yet and need thorough workup. A more real situation can be obtained while experimentally defining the compound conformation with the aid of the X-ray diffraction study combined with pharmacological testing [12]. That is how we have recently registered the first example of conformational polymorphism not known before [13]. While studying *N-*benzyl-4-hydroxy-1-methyl-2,2-dioxo-1*H*-2λ^6^,1-benzothiazine-3-carboxamide (**1**) crystal modifications described before and producing a high analgesic effect [14], it was found that this compound could form two pseudo-enantiomeric forms **A** and **B**, which are mirror images of each other (Figure 1). This fact seems interesting as there is no stereogenic atom in the original molecule. However, due to intramolecular hydrogen-bonding interaction and proper choice of crystallization conditions, it is possible to form crystals based on just one of the mentioned pseudo-chiral conformations **A** or **B** [13].

What is more, analgesic properties of form **A** are about four times higher than those of enantiomer **B** [13], a fact which has stimulated further studies of benzylamide **1** conformers. As the leading role in forming pseudo-enantiomeric forms **A** and **B** belongs to intramolecular hydrogen-bonding interactions, it may be logically assumed that salt formation by 4-OH-group will result in the molecule conformational rearrangement. This report presents the results obtained while studying the structural changes and their effect on biological activity of the compound.

## 2. Materials and Methods

### 2.1. Chemistry

Elemental analysis was performed on a Euro Vector EA-3000 microanalyzer (Eurovector SPA, Redavalle, Italy). Melting points were determined in a capillary using a Stuart SMP10 digital melting point apparatus (Bibby Scientific Limited, Stone, UK). The starting *N-*benzyl-4-hydroxy-1-methyl-2,2-dioxo-1*H*-2λ^6^,1-benzothia-zine-3-carboxamide (**1**) and its pseudo-enantiomeric forms **A** and **B** were prepared by our previously described procedures [14] and [13] respectively. 

### 2.2. Sodium 3-Benzylaminocarbonyl-1-methyl-2,2-dioxo-1*H*-2λ^6^,1-benzothiazin-4-olate Methanol Monosolvate (***2***)

The solution of MeONa in anhydrous methyl alcohol (from metallic Sodium (0.23 g, 0.01 mol) and absolute methanol (20 mL)) is added to the suspension of benzylamide **1** (3.44 g, 0.01 mol) in 20 mL anhydrous MeOH and heated until totally dissolved, filtered and left for 10 to 12 hours under indoor temperature. The crystalline sodium salt methanol monosolvate **2** precipitated was filtered off, and dried in air. Yield: 3.70 g (93%), mp 260–222 °C (decomp.); Analytically calculated (Anal. Calcd.) for C_17_H_15_N_2_NaO_4_S · CH_3_OH: C, 54.27; H, 4.81; N, 7.03; S, 8.05. Found: C, 54.35; H, 4.74; N, 6.94; S, 8.13.

### 2.3. X-ray Structural Analysis of Sodium Salt Methanol Monosolvate (**2**)

The crystals of sodium salt methanol monosolvate **2** (C_17_H_15_N_2_NaO_4_S · CH_3_OH) were triclinic, colorless. At 20 °C: *a* 10.1386(5), *b* 13.9882(7), *c* 14.6555(7) Å; α 63.793(5)°, β 85.510(4)°; γ 86.812(4)°, *V* 1858.6(2) Å^3^, *Z* 2, space group P$\bar{1}$, *d*_calc_ 1.424 g/cm^3^, µ(MoK_α_) 0.230 mm^−1^, *F*(000) 832. The unit cell parameters and intensities of 18,831 reflections (10,794 independent reflections, *R*_int_ = 0.020) were measured on an Xcalibur-3 diffractometer (Oxford Diffraction Limited, Oxford, UK) using MoK_α_ radiation, a Charge Coupled Device (CCD) detector, graphite monochromator, and ω-scanning to 2θ_max_ 60°. The structure was solved by the direct method using the SHELXTL program package (Institute of Inorganic Chemistry, Göttingen, Germany) [15]. The positions of the hydrogen atoms were found from the electron density difference map and refined using the “rider” model with *U*_iso_ = *nU*_eq_ for the nonhydrogen atom bonded to a given hydrogen atom (*n* = 1.5 for methyl and hydroxyl groups, and *n* = 1.2 for the other hydrogen atoms). The structure was refined using *F*^2^ full-matrix least-squares analysis in the anisotropic approximation for non-hydrogen atoms to *wR*_2_ 0.149 for 10,713 reflections (*R*_1_ 0.053 for 7,520 reflections with *F* > 4σ (*F*), *S* 1.066). CCDC 1486591 contains the supplementary crystallographic data for this paper. These data can be obtained free of charge from the Cambridge Crystallographic Data Center [16].

### 2.4. Sodium 3-Benzylaminocarbonyl-1-methyl-2,2-dioxo-1H-2λ^6^,1-benzothiazin-4-olate Monohydrate (***3***)

Sodium hydroxide (0.40 g, 0.01 mol) is added to the suspension of benzylamide **1** (3.44 g, 0.01 mol) in 30 mL H_2_O, heated until totally dissolved, filtered and left for 10 to 12 hours under indoor temperature. The crystalline sodium salt monohydrate **3** precipitated was filtered off, and dried in air. Yield: 3.11 g (81%), mp 281-283 °C (decomp.); Anal. Calcd. for C_17_H_15_N_2_NaO_4_S · H_2_O: C, 53.12; H, 4.46; N, 7.29; S, 8.34. Found: C, 53.04; H, 4.37; N, 7.35; S, 8.42.

### 2.5. X-ray Structural Analysis of Sodium Salt Monohydrate (**3**)

The crystals of sodium salt monohydrate **3** (C_17_H_15_N_2_NaO_4_S · H_2_O) were triclinic, colorless. At 20 °C: *a* 7.930(3), *b* 9.845(3), *c* 11.868(3) Å; α 93.59(2)°, β 92.51(2)°; γ 106.77(3)°, *V* 883.5(4) Å^3^, *Z* 1, space group P$\bar{1}$, *d*_calc_ 1.415 g/cm^3^, µ(MoK_α_) 0.235 mm^−1^, *F*(000) 392. The unit cell parameters and intensities of 5,329 reflections (3,006 independent reflections, *R*_int_ = 0.212) were measured on an Xcalibur-3 diffractometer (Oxford Diffraction Limited, Oxford, UK) using MoK_α_ radiation, a CCD detector, graphite monochromator, and ω-scanning to 2θ_max_ 50°. The structure was solved by the direct method using the SHELXTL program package (Institute of Inorganic Chemistry, Göttingen, Germany) [15]. The positions of the hydrogen atoms were found from the electron density difference map and refined using the “rider” model with *U*_iso_ = *nU*_eq_ for the nonhydrogen atom bonded to a given hydrogen atom (*n* = 1.5 for methyl groups and water, and *n* = 1.2 for the other hydrogen atoms). The structure was refined using *F*^2^ full-matrix least-squares analysis in the anisotropic approximation for non-hydrogen atoms to *wR*_2_ 0.237 for 2,846 reflections (*R*_1_ 0.093 for 784 reflections with *F* > 4σ (*F*), *S* 0.817). CCDC 1486592 contains the supplementary crystallographic data for this paper. These data can be obtained free of charge from the Cambridge Crystallographic Data Center [17].

### 2.6. Pharmacology

#### Analgesic Test

The analgesic properties of the synthesized *N-*benzyl-4-hydroxy-1-methyl-2,2-dioxo-1*H*-2λ^6^,1-benzothiazine-3-carboxamide (**1**) pseudo-enantiomeric forms **A** and **B**, sodium-3-benzylaminocarbonyl-1-methyl-2,2-dioxo-1*H*-2λ^6^,1-benzothiazin-4-olate methanol mono-solvate (**2**), and sodium 3-benzylaminocarbonyl-1-methyl-2,2-dioxo-1*H*-2λ^6^,1-benzothia-zin-4-olate monohydrate (**3**) were studied compared to piroxicam (Jenapharm, Jena, Germany) and meloxicam (Boehringer Ingelheim, Ingelheim am Rhein, Germany) being similar to the structure on the model of the thermal tail-flick procedure in white male rats weighing 180–200 g (Tail Immersion Test) [18]. For this purpose, the rat’s tail tip was immersed in a water bath heated to 54 °С, and the latent period of the tail withdrawal (immersion) expressed in seconds was determined. The analgesic effect (in %) was assessed by the change of the latent period 1 h after introduction of the test substances and the reference drugs. Seven experimental animals were involved to obtain statistically reliable results (the significance level of the confidence interval accepted in this work was *p* ≤ 0.05) in testing each of amides **1**–**3**, the reference drugs and control. Conformers **A** and **B** of benzylamide **1**, piroxicam and meloxicam were introduced orally in the form of fine aqueous suspensions stabilized with Tween-80 in a dose of 20 mg/kg. Sodium salt methanol monosolvate (**2**) and sodium salt monohydrate (**3**) were introduced in the same way in equimolar doses (23 and 22 mg/kg, respectively). The animals of the control group received an equivalent amount of water with Tween-80. 

All biological experiments were carried out in full accord with the European Convention on the Protection of Vertebrate Animals Used for Experimental and Other Scientific Purposes and the Ukrainian Law No. 3447-IV “On protection of animals from severe treatment” (2006) (project ID 3410U14 approved October 15, 2015).

## 3. Results and Discussion

### 3.1. Chemistry

**Scheme 1:**
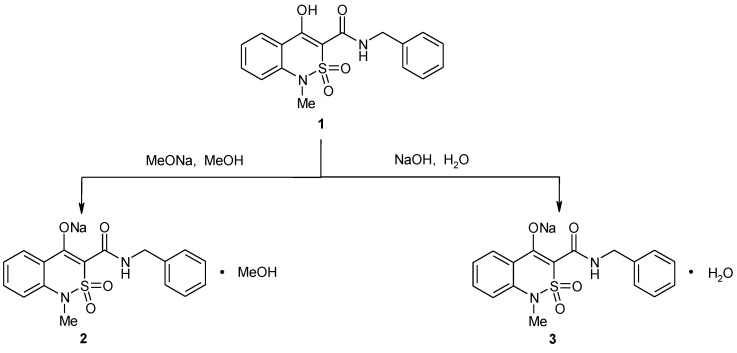
Synthesis of sodium-3-benzylaminocarbonyl-1-methyl-2,2-dioxo-1*H*-2λ^6^,1-benzothiazin-4-olate solvates **2** and **3**.

Synthesis of sodium-3-benzylaminocarbonyl-1-methyl-2,2-dioxo-1*H*-2λ^6^,1-benzothiazin-4-olate was performed with the aid of two methods: *N-*benzyl-4-hydroxy-1-methyl-2,2-dioxo-1*H*-2λ^6^,1-benzothiazine-3-carboxamide (**1**) reaction with an equivalent amount of MeONa or NaOH in methanol or water respectively (Scheme 1). In both cases, the obtained products were colorless crystal substances moderately soluble in hot spirits and water, and with low solubility in cold water. Meanwhile, the synthesized samples melting temperatures proved to be different, which led to the assumption that they had different structures.

In fact, with aid of the X-ray diffraction study it was found that sodium salt methanol monosolvate (**2**) was formed in the first case while monohydrate (**3**) was formed in the second case. Data obtained in the process proved that salt with Na-cation changed *N-*benzyl-4-hydroxy-1-methyl-2,2-dioxo-1*H*-2λ^6^,1-benzothiazine-3-carboxamide conformation considerably.

**Figure 2:**
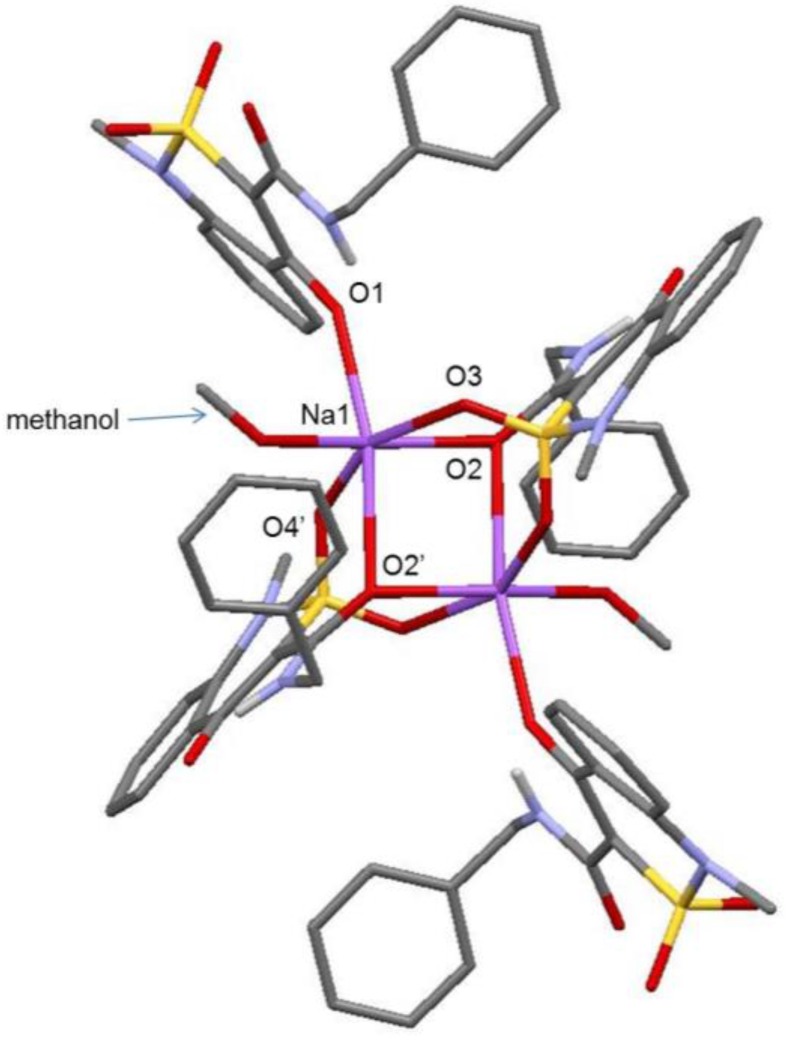
The molecular structure of sodium salt methanol monosolvate (**2**) with numeration of atoms which coordinate Na cation. The hydrogen atoms are omitted for clarity.

**Figure 3:**
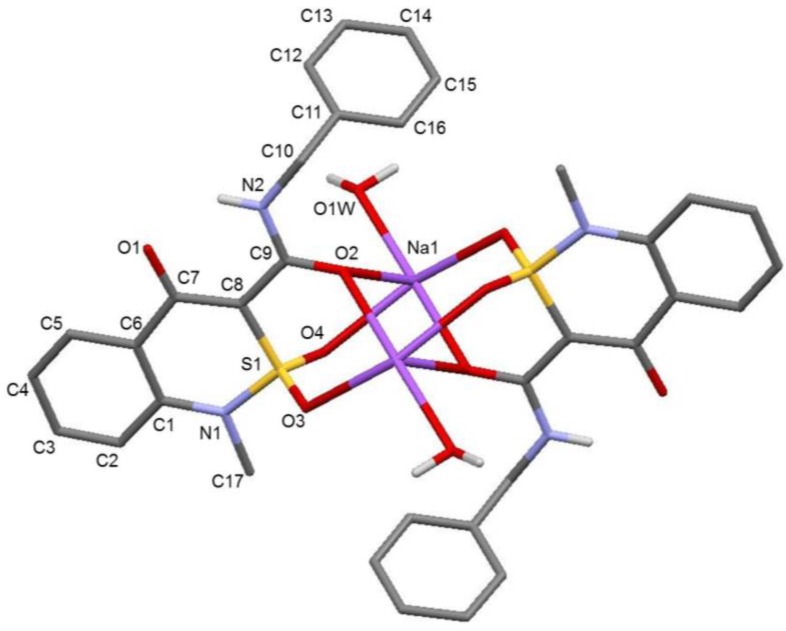
The molecular structure of sodium salt monohydrate (**3**) with numeration of anion atoms of symmetry independent part of complex. The hydrogen atoms are omitted for clarity.

Both compounds have particular location in the crystal in relation to the symmetry center. Two Na-cations and two organic anions (**C** conformer and **D** conformer) with different reciprocal orientation of sulfoxide group and benzyl fragment in relation to the bicycle plane were found in sodium salt methanol monosolvate (**2**) (Figure 2), while only one cation atom was found in the independent part of the elementary cell of monohydrate (**3**) (Figure 3) and, accordingly, one anion molecule (**E** conformer).

Analysis of the molecular anions’ structure in salts **2** and **3** showed that C(7)–O(1) 1.258(2) Å bond in anions **C** and **D** (monosolvate with methanol) and 1.272(6) Å in **E** anion (monohydrate) is longer in comparison with the average value [19] for Csp^2^=O bond and is considerably shorter in comparison to the average value for Сsp^2^–OH (1.362 Å) bond. In addition, it was not possible to localize the hydrogen atom at O(1) atom from electron density difference maps. Consequently, it is possible to suppose that the negative charge is localized on the dehydrated hydroxyl group. It may be supposed that the breakage of O(1)–H…O(2) intramolecular hydrogen bond characteristic of *N-*benzyl-4-hydroxy-1-methyl-2,2-dioxo-1*H*-2λ^6^,1-benzothiazine-3-carboxamide (**1**) [13] and the presence of the negative charge lead to carbamide fragment rotation (torsion angle С(7)–С(8)–С(9)–N(2) 5.7(2)° in **С**, −7.7(3)° in **D** and 5.5(6)° in **Е**) and forming of the N(2)–H…O(1) charge-strengthened intramolecular hydrogen bond (H…O 1.97 Å, N–H…O 136° in **C** anion, H…O 1.97 Å, N–H…O 134° in **D** anion and H…O 1.95 Å, N–H…O 136° in **Е** anion). Consequently, the sulfur atom of the sulfo group, which was pseudo-chiral in **A** and **B** conformers [13] because of N–H…O=S hydrogen bond forming, loses this quality. Besides, absence of intramolecular hydrogen N–H…O=S bond and participation of sulfo group oxygen atoms in complex formation with Na-cation lead to considerable changing of dihydrothiazine heterocycle conformation. In fact, in the case of monosolvate with methanol, the dihydrothiazine cycle adopts a conformation in between the twist-boat and sofa (puckering parameters [20] are: S = 0.36, Θ = 59.1°, Ψ = 2.8° in **С** anion and S = 0.37, Θ = 42.5°, Ψ = 23.0° in **D** anion), but flattens considerably. Under these conditions, atoms S(1) and С(8) in **С** and **D** anions deviate to different sides from mean-square plane of the cycle remaining atoms (deviation amounts to 0.51 Å and 0.33 Å, respectively, in **С** and −0.47 Å and −0.18 Å, respectively, in **D**).

**Figure 4:**
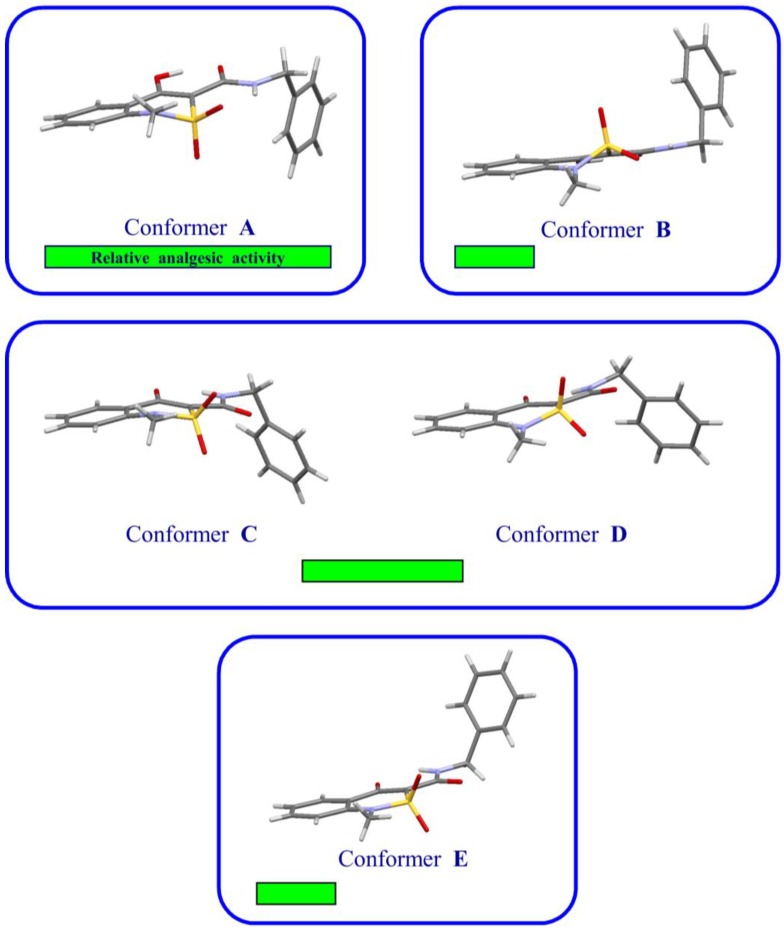
Highly active (**A**) and low active (**B**) conformers of benzylamide (**1**) (experimental data); highly active (**C**) and low active (**D**) conformers of sodium salt methanol solvate (**2**) (presumably); low-active conformer (**E**) of sodium salt hydrate (**3**) (experimental data). Sodium atoms and molecules of the solvents in the conformers (**C**–**E**) are not specified.

In the case of monohydrate sodium salt formation, the dihydrothiazine heterocycle is planar with precision 0.02 Å. Under these conditions, the aromatic cycle of benzyl fragment orthogonally orientated in respect to the carbamide group plane (torsion angles C(9)–N(2)–C(10)–C(11) 84.0(3)° in **С** 86.2(2)° in **D** and –80.2(6)° in **Е**) is orientated to different sides in respect to benzothiazine fragment. Therefore, when sodium salt forms, we obtain *N-*benzyl-4-hydroxy-1-methyl-2,2-dioxo-1*H*-2λ^6^,1-benzothiazine-3-carboxamide (**1**) conformation, which is very close to active form **A** but has flattened dehydrated cycle (conformer **С**), and two new forms: conformer **D** with diversely orientated sulfo group and aromatic cycle of benzyl fragment and conformer **E** with planar bicyclic fragment. It can be supposed that the difference in anion structure results from the peculiarities of the complex with Na cation formation (Figure 4).

**Figure 5:**
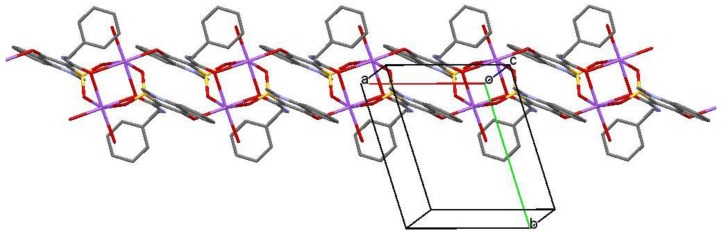
Infinite chain (1D polymer) formed by sodium salt monohydrate (**3**) along [100] crystallographic direction in the crystal phase. The hydrogen atoms are omitted for clarity.

**Figure 6:**
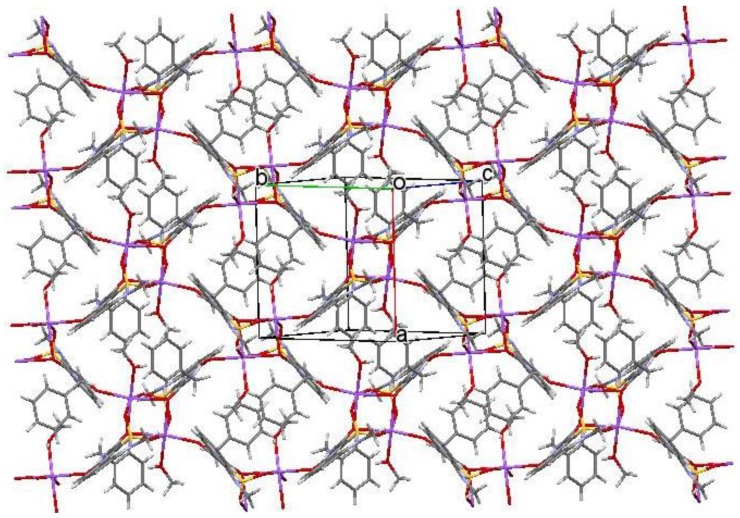
Layer (2D polymer) formed by sodium salt methanol monosolvate (**2**) in the crystal phase. The hydrogen atoms are omitted for clarity.

Crystal structure analysis showed that coordinational polyhedron of Na cation represents a distorted octahedron in monosolvate with methanol as well as in monohydrate. In both structures, two Na-cations have chelate type connections with two bridge anions and are coordinated by atoms O(2), O(3) and O(4). Besides, every Na atom is coordinated by an O(1) atom of the third anion, which bonds Na-anion pairs, and by a solvent molecule. However, the location of anion coordinating Na cation in non-chelate type is different in structures **2** and **3**. In the case of monohydrate (structure **3**), this anion forms a stacking dimer of the type “head to tail” with “chelate” anion. As a result, a 1D polymer (Figure 5), which is essentially a chain of staking dimers interconnected by a pair of Na cations every one of which is additionally coordinated by a water molecule, forms in the crystal. However, in case of monosolvate with methanol, the anion, which additionally coordinates Na atom, is located orthogonally to the “chelate” anion which results in 2D polymer (Figure 6) in the crystal.

### 3.2. Evaluation of the Analgesic Activity

Testing of the analgesic activity of the two pseudo-enantiomeric forms of the original *N-*benzyl-4-hydroxy-1-methyl-2,2-dioxo-1*H*-2λ^6^,1-benzothiazine-3-carboxamide (**1**) and its sodium salt solvates **2** and **3** was performed simultaneously under the same conditions and in equimolar dozes. Meanwhile it was found that sodium 3-benzylaminocarbonyl-1-methyl-2,2-dioxo-1*H*-2λ^6^,1-benzothiazin-4-olate methanol monosolvate (**2**) analgesic properties level is about two times lower than that of highly active (**A**) conformer of benzylamide **1**, but two times higher than that of both its low active (**В**) conformer and monohydrate form of sodium salt (**3**) (Table 1).

A more detailed analysis and comparison of the X-ray diffraction study results and pharmacological research reveals some very interesting structural-biological relationships. Conformer **C** of sodium-3-benzylaminocarbonyl-1-methyl-2,2-dioxo-1*H*-2λ^6^,1-benzo-thiazin-4-olate methanol monosolvate (**2**) happens to be structurally close to highly active conformer **A** of benzylamide (**1**) (Figure 4). That is why it can be assumed to be the component providing the average level of sodium salt methanol solvate (**2**) analgesic activity in general. But conformer **D** located next to it in the crystal is most likely to be much less active and represents kind of a “ballast”. Here (analogically to low-active conformer **В**), the sulfoxide group is located in a position which is “uncomfortable” for the closest contact with the biological target of the position. In other words, strict complementarity between the compound and receptor happens to be broken, even though only partially. Consequently, conformer **D** is not to be expected to develop maximum analgesic effect. Spatial structures of low active **В** and **Е** conformers are also quite similar (Figure 4), which can explain their practically identical analgesic effect.

## 4. Conclusions

This article contains a report on the results of the complex research on synthesis, real spatial structure defining and pharmacological testing of *N-*benzyl-4-hydroxy-1-methyl-2,2-dioxo-1*H*-2λ^6^,1-benzothiazine-3-carboxamide conformational isomers. It was proved that salt formation causes considerable conformational restructuring of the original molecule. Biological tests showed that different conformers develop analgesic activity which may differ several times. According to the results obtained, the most biologically active conformer of *N-*benzyl-4-hydroxy-1-methyl-2,2-dioxo-1*H*-2λ^6^,1-benzothiazine-3-carboxamide was identified.

## Figures and Tables

**Table 1:** The analgesic activity of pseudo-enantiomeric forms of benzylamide (**1**), solvates of sodium salts **2** and **3**, and reference drugs.

Entry	Product	Conformer	Latent Period in 1 h after Introduction of the Compounds (s) ^a^	Change of the Latent Period, Compared to Control (%)
1	**1**	**A**	15.98 ± 0.85	+111.9
2	**1**	**B**	9.88 ± 0.72	+31.1
3	**2**	**C–D (1:1)**	12.27 ± 0.81	+62.7
4	**3**	**E**	9.86 ± 0.70	+30.8
5	Meloxicam	-	11.27 ± 0.83	+49.5
6	Piroxicam	-	9.45 ± 0.74	+25.3
7	Control	-	7.54 ± 0.82	-

^a^ All results from biological tests were analyzed statistically using Student’s *t*-test. Effects were regarded as statistically significant at *p* ≤ 0.05.
